# Fermentation quality, microbial dynamics, and rumen degradability of total mixed ration silages containing *Manihot* sp’ foliage

**DOI:** 10.1007/s11250-026-04934-w

**Published:** 2026-02-26

**Authors:** Alex Rodrigues de Sousa, Marcos Jácome de Araújo, Otávio Tavares Medeiros, Tairon Pannunzio Dias-Silva, Antonio Leandro Chaves Gurgel, Rafael de Souza Miranda, Leilson Rocha Bezerra, Luís Carlos Vinhas Ítavo, Gelson dos Santos Difante, Ricardo Loiola Edvan

**Affiliations:** 1 Department of Animal Science, Federal University of Piauí, Bom Jesus, Piaui Brazil; 2 Department of Animal Science, Technology Center of Health and Animal Production, Federal University of Campina Grande, Patos, Paraiba Brazil; 3 College of Veterinary Medicine and Animal Science, Federal University of Mato Grosso do Sul, Mato Grosso do Sul, Campo Grande, Brazil

**Keywords:** Bacteria, Effluent losses, Pornunça, Ruminal degradation, Semiarid, Yeasts

## Abstract

Pornunça (*Manihot* sp.) is a potential forage for feeding ruminants in drylands worldwide due to its drought tolerance and favorable nutritional value. This study aimed to evaluate the quality, fermentative profile, losses, and ruminal degradability of total mixed ration (TMR) silages containing pornunça foliage as a fiber source. The treatments consisted of TMR silages with different forage-to-concentrate ratios (50:50, 60:40, 70:30, 80:20, and 100:0, DM basis), arranged in a completely randomized design with five replications. Ruminal degradability (DEG) was evaluated using three rumen-cannulated sheep (46.0 ± 2.30 kg). Exclusive ensiling of pornunça negatively affected fermentative characteristics, increasing gas and effluent losses, reducing dry matter recovery (DMR), and numerically increasing yeast and enterobacteria counts. The highest DMR was observed in the 50:50 silage (916 g/kg), likely due to lower fermentative losses. Silages with a higher concentrate proportion (50:50 and 60:40) showed greater concentrations of non-fibrous carbohydrates and higher production of organic acids, especially lactic and acetic acids. All silages exhibited propionic, acetic, and butyric acid concentrations below the maximum recommended limits for good-quality silage. These characteristics resulted in a higher soluble fraction ‘*a*’ and the highest effective DEG of DM and neutral detergent fiber (NDF) in the 50:50 silage. In contrast, the 100:0 silage showed a high fiber concentration, which likely contributed to its lower “*a + b*” fraction and reduced effective DM degradability. Despite the superior quality of the silage with a 50:50 forage-to-concentrate ratio, pornunça demonstrates high potential as a sustainable forage source for semiarid regions.

## Introduction

Small ruminant farming is one of the most important socioeconomic activities for populations of semiarid regions, where native rangelands represent an important feed resource for livestock. However, this type of vegetation does not fully meet the nutritional requirements of animals throughout the year, resulting in low performance rates. Therefore, the seasonality of forage production represents one of the biggest challenges to ruminant farming in these regions (Carvalho et al. 2017).

Given this scenario, growing plants adapted to semiarid conditions can maintain the balance in feed supply throughout the year, reducing the problem of forage supply seasonality. Therefore, it becomes necessary to carry out research with native species, especially regarding their nutritional value, and their potential to be ensiled and used in animal feeding, aiming at the sustainability of the production system (Santos et al. 2010; Pereira et al. 2012).

Pornunça (*Manihot* sp.) is a species belonging to the *Euphorbiaceae* family, is a natural hybrid between cassava (*Manihot esculenta*) and wild cassava (*Manihot pseudoglaziovii*), which stands out for its high production and retention of leaves, high tolerance to harvest and water stress, high starch accumulation, high crude protein content, high sprouting capacity, and high nutritional value of the hay and silage produced (Voltolini et al. 2010; Alencar et al. 2015; Carvalho et al. 2017).

Pornunça presents satisfactory forage yield, considering the Brazilian semiarid environment (Alencar et al. 2019). The forage production of *Manihot* sp. in the semi-arid region varied from 3.5 t/ha in the first year, with only one cut, to 27.2 t/ha of original material in the third year with two annual cuts (Silva et al. 2009). Considering the dry matter (DM) yield, Ferreira et al. (2009) obtained yields of 468 and 178 kg DM/ha for the first and second cut, respectively. On the other hand, Vasconcelos et al. (2010) observed DM yield ranging from 218 to 529 kg/ha for unfertilized and fertilized area with bovine digesta, respectively.

Ensiling is an important practice that allows for the surplus forage produced in the rainy season to be better used as a reserve for the drought periods. Ensiling plants of the *Euphorbiaceae* family, besides being an important feed alternative to minimize the effects of drought, prevents the intoxication of animals through cyanogenic glycosides, because they are inactivated (Backes et al. 2014; Sena et al. 2014). However, these plants present limitations for ensiling because they have low DM contents and high crude protein contents (above 150 g/kg DM) which may constitute in buffering capacity and impair the preservation process, causing a reduction in the pH drop rate of this material in the form of silage and increasing forage losses during the preservation period (Nascimento et al. 2016; Borreani et al. 2018).

The use of forage plants adapted to semiarid regions, along with the practice of ensiling, increases the forage allowance, especially during the drought season of the year, and can make sheep farming a sustainable activity (Carvalho et al. 2017). In this context, Pornunça represents an alternative feed for ruminants due to its abundance and nutritional characteristics, as well as its productive potential (Amorim et al. 2023). However, for being a plant of the genus *Manihot* contains cyanogenic glycosides that generate hydrocyanic acid (HCN) when hydrolyzed, which can be toxic to the animal depending on the amount ingested (Beltrão et al. 2015). The conservation of Pornunça as silage reduces HCN concentration and is considered a safe strategy to feed ruminants.

The Pornunça silage has been widely used in the breeding of goats and sheep in the Brazilian semi-arid. This silage is usually made with the aerial part of the plant (young leaves and tender stems), chopped up to an average particle size of approximately 2.0 cm in forage machine, homogenized and compacted in plastic-drums silos with a capacity of 200 L, with removable lid sealed with a metal ring at a density of 660 kg fresh matter/m^3^ and stored for 60 days, especially in smallholder farming systems. The silage can be made from Pornunça alone, but it’s also common to mix Pornunça with other forages for improved nutritional value and to address specific needs, such as cactus pear (*Opuntia* sp.; Silva et al. 2021), old man saltbush (*Atriplex nummularia*; Voltolini et al. 2019).

Under the experimental conditions, Carvalho et al. (2017) evaluated the effects of inclusion of Pornunça silage (500 g/kg DM) in the diet of feedlot lambs and observed consumption of DM and crude protein of 710 and 670 g/day, respectively, and average daily gain of 208.2 g/day. Later, Amorim et al. (2023) demonstrate that the hay of Tifton-85 could be totally replaced by the silage of Pornunça, in diets for lambs in confinement. The use of silages made from forage plants adapted to the semi-arid conditions such as Pornunça can yield satisfactory results regarding quantitative and qualitative traits of meat from feedlot-finished lambs, as observed by Campos et al. (2017) which found that old man saltbush and Pornunça were better silages.

Total mixed ration (TMR) ensiling, originally developed in Asian countries, particularly Japan, has been adopted as an alternative strategy for feed preservation and for the utilization of food industry coproducts (Bueno et al. 2020). This technique enables the use of ingredients with high buffering capacity and low DM and soluble carbohydrate contents through dietary complementarity, ensuring adequate fermentation (Hu et al. 2015). However, given the potential losses during ensiling and the high cost of TMR ingredients, a thorough understanding of ingredient composition, ration balancing, and ensiled microbiota is essential to maximize nutrient conservation (Bueno et al. 2020).

Pornunça has shown promising potential as a raw material for silage production, having been studied both in isolation (Campos et al. 2017; Carvalho et al. 2017) and in association with other forages (Silva et al. 2021; Gomes et al. 2023; Godoi et al. 2024). However, studies evaluating its use in the form of TMR silage are still scarce. Thus, considering the fermentative and economic advantages of TMR silage (Bueno et al. 2020) and the potential of Pornunça as a forage resource for semiarid regions, further research is needed to evaluate this feeding strategy.

The use of TMR silage with locally available ingredients is a key strategy to ensure consistent ruminant feed throughout the year, particularly in regions with seasonal forage variability (Du et al. 2020). Effective application requires understanding the fermentation dynamics, including changes in ingredient composition, microbial activity, and fermentation products, and their impact on animal performance. Forage-to-concentrate ratios (F: C) critically affect TMR silage quality by influencing substrate availability and buffering capacity. Higher concentrate proportions increase fermentable carbohydrates, favor lactic acid bacteria, accelerate pH decline, and reduce gas and effluent losses, enhancing dry matter and energy recovery. In contrast, higher forage proportions increase fiber and buffering capacity, slowing fermentation and raising nutrient losses (Kung Jr et al. 2003). Optimizing the F: C ratio is therefore essential to improve fermentation efficiency and preserve nutrients in TMR silages.

In this context, it was hypothesized that ensiling TMR from the incorporation of concentrated feed to the Pornunça (fresh fiber source), will promote improvements in the fermentative process, reducing losses and allowing the preservation of the silage nutritional value. Therefore, this study aimed to evaluate the fermentation quality, microbial population, aerobic stability, and rumen degradability of TMR silages containing Pornunça at different forage-to-concentrate ratios.

## Materials and methods

### Experiment location

The experiment was conducted at the Federal University of Piauí (CPCE-UFPI), Bom Jesus, Piauí, Brazil, which is located at latitude 9º4’28” South, longitude 44º21’31” West, and altitude of 277 m. The region presents summer rainfalls and dry winter, being classified as Aw, according to the Köppen classification, described by Alvares et al. (2014), with minimum temperature of 18 °C and maximum of 36 °C, and average annual rainfall precipitation of 900 mm.

### Experimental design and total mixed ration (TMR) silage preparation

The experimental design was completely randomized with five treatments and five replications. Four silages were made from TMR in different forage-to-concentrate ratios (F: C; 50:50, 60:40, 70:30, 80:20, and 100:0; DM basis). The concentrate used was based on ground corn and soybean meal and the rations were iso-protein, formulated to meet the nutritional requirements of lambs weighing 30 kg of BW with a daily weight gain of 200 g (NRC 2007). The formulations and chemical compositions of the TMR before ensiling are given in Table 1.

**Table 1:** Ingredient and chemical composition of total mixed ration at the time of ensiling

Item	Pornunça (Manihot sp.)		Ground corn		Soybean meal	
Chemical composition (g/kg DM)						
Dry matter (g/kg fresh weight)	244		845		849	
Ash	71.0		13.5		67.3	
Crude protein	168		95.0		529	
NDIP (g/kg crude protein)^a^	264		139		115	
ADIP (g/kg crude protein)^b^	95.6		35.8		35.6	
Ether extract	25.4		37.6		26.4	
Total carbohydrates	735		854		377	
Neutral detergent fiber^d^	486		131		159	
Acid detergent fiber	368		49.2		91.3	
Non-fiber carbohydrates	250		723		218	
Cellulose	262		35.2		75.7	
Hemicellulose	117		81.5		67.9	
Acid detergent lignin	107		14.0		15.6	
	Forage-to-concentrate ratio (DM basis)					
	50:50	60:40	70:30	80:20		100:0
Ingredient proportion (g/kg DM)						
Pornunça	500	600	700	800		1000
Ground corn	416	333	250	167		-
Soybean meal	84.0	67.0	50.0	33.0		-
Chemical composition (g/kg DM)						
Dry matter (g/kg fresh weight)	378	341	310	284		244
Ash	46.7	51.6	56.4	61.3		71.0
Crude protein	168	168	168	168		168
NDIP (g/kg crude protein)^a^	199	213	226	238		264
ADIP (g/kg crude protein)^b^	65.7	71.7	77.6	83.6		95.6
Ether extract	30.6	29.5	28.5	27.4		25.4
Neutral detergent fiber^c^	311	346	381	416		486
Acid detergent fiber	212	244	275	306		368
Total carbohydrates	754	751	747	743		735
Non-fiber carbohydrates	444	405	366	328		249
Cellulose	152	174	196	218		262
Hemicellulose	98.1	102	106	109		117
Acid detergent lignin	60.6	69.8	79.1	88.4		107

DM: dry matter

^a^ NDIP: neutral detergent insoluble protein

^b^ ADIP: acid detergent insoluble protein

^c^ Neutral detergent fiber using a heat stable amylase without the use of sodium sulfite and not corrected for ash and protein

The soil at the experimental site was classified as Typic Haplustox (Soil Survey Staff) and Typical Dystrophic Yellow Latosol according to the Brazilian Soil Survey. Pornunça was grown in an area owned by UFPI and 12 months after planting, the leaves and the most tender stems was harvested to make the silage. The fresh plant was mechanically processed in a stationary forage chopping machine (model 30648-2 Garthen^®^, Santa Catarina, Brazil) equipped with a 2 cm sieve. Part of the fresh plant material was manually mixed with the concentrate ingredients to prepare the TMR while the other part was used to make the silage that had 100 g/100 g of Pornunça (100:0).

For this study, experimental silos made from polyvinyl chloride (PVC) with a capacity of 3 kg were utilized. These silos had a diameter of 10 cm and a length of 40 cm. To ensure adequate compaction, the material was compacted using wooden sticks until a density of 613 ± 51 kg/m^3^ was achieved. The silos were equipped with a Bunsen-type valve adapted to the lid, allowing for the release of fermentation gases. Sand (0.5 kg) was deposited in the bottom of each silo, which was separated from the ensiled material by a layer of cotton fabric, to determine the amount of effluent produced.

### Analysis of fermentative parameters

For the fermentation profile analyses, seventy-five silos were used, of which fifteen silos were opened at 1, 15, 30, 60, and 90 days after closure. The other variables were evaluated using the silos that were opened at 90 days. From the material of each silo (replication), composite samples were formed by treatment, from which subsamples were randomly collected for the evaluation of chemical composition and fermentative parameters. Microbial populations were assessed in fresh samples, while the remaining material was stored at -20 °C until analysis.

The pH determination in distilled water was performed in triplicate by collecting 25 g of samples of the ensiled material of each treatment and adding 100 mL of water according to the methodology described by Bolsen et al. (1992). The NH_3_-N content (g/kg total nitrogen; TN) was analyzed according to the methodology described by Licitra et al. (1996). The buffering capacity (BC) was determined according to the methodology of Mizubuti et al. (2009), using 10 to 20 g of macerated silage with 250 mL of distilled water. The BC was calculated by the equation:

$$BC\, = \,0.1\,{\rm{ }} \times \,{\rm{ }}[({V_1}\,-\,{\rm{ }}{V_2})\,{\rm{ }}/\,DW]\, \times \,100,$$

where BC = buffering capacity in mg NaOH equivalent/100 g DM; 0.1 = NaOH Normality; V_1_ = volume of NaOH spent to change the pH of the sample from 4.0 to 6.0; V_2_ = volume of NaOH spent to change the pH of the blank from 4.0 to 6.0; DW = dry sample weight = [(sample weight × DM) /100].

For the determination of concentrations of organic acids (lactic, acetic, propionic and butyric) 10 g of each silage were weighed (in triplicate), then 90 ml of distilled water was added, homogenized in a blender for 1 min and filtered in a syringe filter with pore of 0.22 μm. Subsequently, a 10 ml sample was taken from the filtrate, which was placed in tubes to be centrifuged and 1.0 mL of metaphosphoric acid and two drops of the 50% H_2_SO_4_ were added then the solution formed was centrifuged for 15 min at 13,000 × g. After this process, the supernatant was collected in Eppendorf tubes, and frozen for determination of organic acid concentrations using high performance liquid chromatography (HPLC; SHIMADZU, SPD-10 A VP) technique (Siegfried et al. 1984). The HPLC apparatus was equipped with an Ultraviolet Detector using an Aminex HPX-87 H column (BIO-RAD, CA, USA) with the mobile phase containing 0.005 M H_2_SO_4_, flow rate of 0.6 ml/min and wavelength of 210 nm. All procedures of organic acid analysis were performed in the Animal Nutrition and Soil Analysis Laboratories of UFPI.

Dry matter losses in silages, in form of gases and effluents, were quantified by weight difference according to methodologies proposed by Jobim et al. (2007).

Gas losses were obtained by use of the following equation:

$$GL\, = \,\left[ {\left( {WSc\,-\,WSo} \right)\,/\,\left( {FMc\, \times \,DMc} \right)} \right]\, \times \,100,$$

where: GL = losses through gas during storage (g/kg DM); WSc = weight of the silo at closure (kg); WSo = weight of the silo at opening (kg); FMc = forage mass at closure (kg); DMc = forage dry matter content at closure (g/kg).

The losses through effluent were calculated based on the weight difference of the sand related to the fresh forage mass at closure by using the following equation:

$$EL\, = \,\left[ {\left( {EWo\,-\,St} \right)\,-\,\left( {EWc\,-\,St} \right)} \right]{\rm{ }}\,/\,FMc\, \times \,100,$$

where: EL = effluent losses (kg/ton silage); EWo = empty silo weight + sand weight at opening (kg); St = silo tare; EWc = empty silo weight + sand weight at closure (kg); FMc = forage mass at closure (kg).

To estimate the DM recovery (DMR), the following equation was adopted:

$$DMR\, = \,\left( {FMo\, \times \,DMo} \right)\,/\,\left( {FMc\, \times \,DMc} \right)\, \times \,100,$$

where: DMR = dry matter recovery rate (g/kg); FMo = forage mass at opening (kg); DMo = silage dry matter content at opening (g/kg DM); FMc = fresh forage mass at closure (kg); DMc = forage dry matter content at closure (g/kg DM).

### Microbial population

Silage samples from each treatment were sampled for population counts of yeasts, molds, enterobacteria (ENT) and lactic acid bacteria (LAB). Microbiological evaluation was performed according to the recommendations of Gonzáles and Rodrigues (2003) by collecting 25 g of fresh silage at 90 days of fermentation. Microbial populations were quantified using selective culture medium for each microbial group, as follows: Rogosa Agar (Difco™, MERCK, Darmstadt, Germany), for counting LAB after incubation for 48 h in an oven at 37 °C; Brilliant Green Bile Agar medium (Difco™, MERCK, Darmstadt, Germany) for counting ENT after incubation for 24 h at 35 °C; Potato Dextrose Agar medium (MERCK, Darmstadt, Germany) acidified with 1% tartaric acid to counting molds and yeasts after 48 h of incubation at room temperature.

### Aerobic stability

The silos were opened disregarding the surface layer and mixing the silage content of each treatment in a plastic container that allowed good homogenization and decompression of the material, which favored the penetration of air.

The samples were kept in a climate-controlled environment, with the temperature maintained at 25 °C using an air-conditioning system. The internal silage temperature was measured every hour, during 96 h of exposure to air, using a digital thermometer (INCOTERM^®^) inserted at 10 cm from the center of the silage mass as proposed by Kung Jr et al. (2003). Approximately 50 g of the mass from the same sample were taken at intervals of 8 h (three samples per day), during four days of exposure to air, for determination of pH values. The break of aerobic stability was considered when the temperature of the silage was 2 °C above ambient temperature, according to Taylor et al. (2002).

### Chemical composition

The pre-dried samples were ground in a Willey-type mill (1 mm screen, Wiley Mill, Arthur H. Thomas, Philadelphia, PA, USA), and placed in closed plastic containers for further chemical analyses. The DM contents (Method: 930.15), crude protein (CP) (*N* × 6.25; Method: 981.10), mineral matter (MM) (Method: 942.05), and ether extract (EE) (Method: 920.29) were determined according to AOAC (1990). Neutral detergent fiber (NDF) and acid detergent fiber (ADF) were analyzed according to Van Soest et al. (1991), and the procedure was modified in this study using non-woven textile (NWT) bags (100 g/m^2^). For analysis of NDF, samples were treated with α-amylase, and no sodium sulfite (aNDF) was used, and the residue wasn’t corrected for the residual ash and residual nitrogen content. Neutral detergent insoluble protein (NDIP) and acid detergent insoluble protein (ADIP) were determined in the residues of NDF and ADF, respectively, according to methodology described by Sniffen et al. (1992). For the determination of acid detergent lignin (ADL), ADF residues were subjected to digestion with 72% H_2_SO_4_ solution.

Total carbohydrates (TC) were obtained by the equation: 100 - (g/kg CP + g/kg EE + g/kg ashes), and non-fiber carbohydrates (NFC) by the difference between TC and NDF, as proposed by Sniffen et al. (1992). Total digestible nutrients (TDN) were estimated according to the equation described by Undersander et al. (1993): TDN (g/kg DM) = 889 - [g/kg, ADF (g/kg, DM) × 0.779].

### In situ rumen degradability

Ruminal degradation was determined by the incubation of dried and ground silage samples (2-mm screen) in NWT bags (Valente et al. 2011). The bags were placed into a nylon bag and suspended below the particulate mat layer in the rumen of three rumen-fistulated sheep (46.0 ± 2.30 kg BW) fed a diet containing 60% roughage (Elephant grass) and 40% concentrate (corn ground, soybean meal and mineral supplement).

The bags were incubated for 0, 6, 12, 24, 48, 72 and 96 h in the rumen. The bags were placed in reverse order, with five replications to be all removed from the rumen at the same time and immediately rinsed in cold water, and frozen. After completing all incubations, bags were thawed and washed under running water until the water was clear. Zero-hour bags were not incubated in the rumen but were included in the washing procedure. Washed bags and contents were dried at 55 ◦C for 72 h and after that they were placed in an oven at 105 °C for 2 h and weighed. The incubation residues were ground in a Willey type mill with a 1-mm-mesh sieve for subsequent determination of DM and NDF.

The disappearance of DM and NDF were calculated by the difference of bag weights before and after incubation, based on the DM. The DM disappearance data were fit to the equation proposed by Ørskov and Mcdonald (1979): *Dt* (g/kg) = [(*a* + *b*) × (1 – *e*^-*ct*^)], where *Dt* is the cumulative amount degraded at time *t*, the parameters *a*, *b* and *c* represent the fraction disappearing immediately, the fraction potentially degradable and the fractional degradation rate of *b*, respectively. Thus (*a + b*) is the potential degradability of the material.

The equation *ED* = a + b × c/(c + k) was used to calculate DM and NDF effective degradability (*ED*). In this equation, k represents the flow rate of particles out of the rumen. This parameter is dependent on the level of DM intake. Feed passage rates of 20, 50 and 80 g/kg/hour were considered for the levels: low, medium, and high intake, according to the model proposed by Ørskov and McDonald (1979).

The non-degraded residue of NDF was estimated using the model of Mertens and Loften (1980): *Rt* (g/kg) = [*b* × (1 - *e*^-*ct*^) + *U*], where *Rt* is the non-degraded residue of NDF in time *t*; *b* is potentially degradable insoluble fraction (g/kg) and *u* is the undegradable fraction (g/kg). After the adjustments of the NDF degradation equation, it proceeded to the standardization of fractions, as proposed by Waldo et al. (1972), using the equations:

*SB* (g/kg) = {[*b* / (*b* + *u*)] × 100} and *SU* (g/kg) = {[*u* / (*b* + *u*)] × 100}, where: *SB* is the standardized potentially degradable fraction (g/kg); *SU* is the standardized undegradable fraction (g/kg); *b* is potentially degradable insoluble fraction (g/kg) and *u* is the undegradable fraction (g/kg). In the calculation of the *ED* of NDF, it was considered the following model: *ED* (g/kg) = (*SB* × *c*) / (*c* + *k*), where *SB* is the standardized potentially degradable fraction (g/kg).

After the data were adjusted and using the disappearance value obtained at time zero of degradation (*a*), the colonization time (lag time) for DM and NDF was estimated, according to Goes et al. (2010): Lag time (h)= [-ln(*a*’-*a*-*b*) / *c*], where the parameters *a*, *b*, and *c* were estimated by the Gauss Newton algorithm.

### Statistical analysis

All data were analyzed using mixed models and the MIXED procedure of SAS (SAS Inst. Inc., Cary, NC). Residuals were plotted against predicted values and were used to check the model assumptions of homoscedasticity, independence, and normality of errors. Data was considered an outlier and removed from the database when the studied residuals were outside the range of ± 2.5. Means were compared using the LSMEANS procedure with Tukey–Kramer adjustment and differences were considered significant at *P* ≤ 0.05, using the following model:

$${Y_{ij}}\, = \,\mu \, + \,{S_i} + {\rm{ }}{e_{ij}},$$

where: Y_ij_ = dependent variable; µ = overall mean; Si = fixed effect of silage type (S = 1.5); e_ij_ = random error associated with each observation.

For the evaluation of chemical composition data, a completely randomized design was considered. For the fermentative profile, a 5 × 5 factorial arrangement was adopted, with five silages and five evaluation periods (opening days). When the interactions were significant, the degrees of freedom were sliced (SLICE option). The effects of fermentation period on pH were evaluated using orthogonal contrasts to determine linear or quadratic effects. The contrasts were significant when *P* ≤ 0.05. The effect of different silages (F: C) on pH was compared using the LSMEANS procedure with Tukey–Kramer adjustment and differences were considered significant at *P* ≤ 0.05, using the following model:

$${Y_{ijk}}\, = \,\mu \, + \,{S_i}\, + \,{P_j}\, + \,({S_i}{P_j})\, + \,{\rm{ }}{e_{ijk,}}$$

where: Y_ijk_ = dependent variable; µ = overall mean; S_i_ = fixed effect of silage (S = 1…5); P_j_ = fixed effect of fermentation period (*P* = 1…90 days); S_i_P_j_ = fixed effect of S × P interaction; e_ijk_ = random error associated with each observation.

For the aerobic stability data, a 5 × 4 factorial arrangement was adopted, with five treatments (TMRs) and four days of air exposure and five replications (five silos). When the interactions were significant, the degrees of freedom were sliced (SLICE option). Temperature and pH were also evaluated as a function of hours of air exposure, with the hours being considered as a repeated measure over the time (REPEATED option), considering the model below:

$${Y_{ijk}}\, = \,\mu \, + \,{S_i}\, + \,{\rm{ }}{H_j}\, + \,({S_i}{H_j})\, + \,{e_{ijk}},$$

where: Y_ijk_ = dependent variable; µ = overall mean; S_i_ = silage effect (S_i_ = 1…5); H_j_ = fixed effect of air exposure h (H = 24…96 h); S_i_H_j_ = fixed effect of S × H interaction; e_ijk_ = random error associated with each observation. Means were compared using the LSMEANS procedure with Tukey–Kramer adjustment and differences were considered significant at *P* ≤ 0.05.

The data of microbial populations (LAB, yeasts, ENT, and molds) were analyzed descriptively, presented in logarithmic units (log^10^ CFU/g silage), and displayed as graphs.

The in-situ degradability test was performed in randomized block design, in split-plots over time, where the three animals represented blocks; silage represented the plots; and the seven times of incubation of feed on the rumen represented the subplots. The data used (observed) for estimation of rumen degradation parameters were analyzed by the interactive method using the SAS NLIN procedure for non-linear models. Means were compared using the LSMEANS procedure with Tukey–Kramer adjustment and differences were considered significant at *P* ≤ 0.05.

## Results

### Fermentation profile

**Fig. 1:**
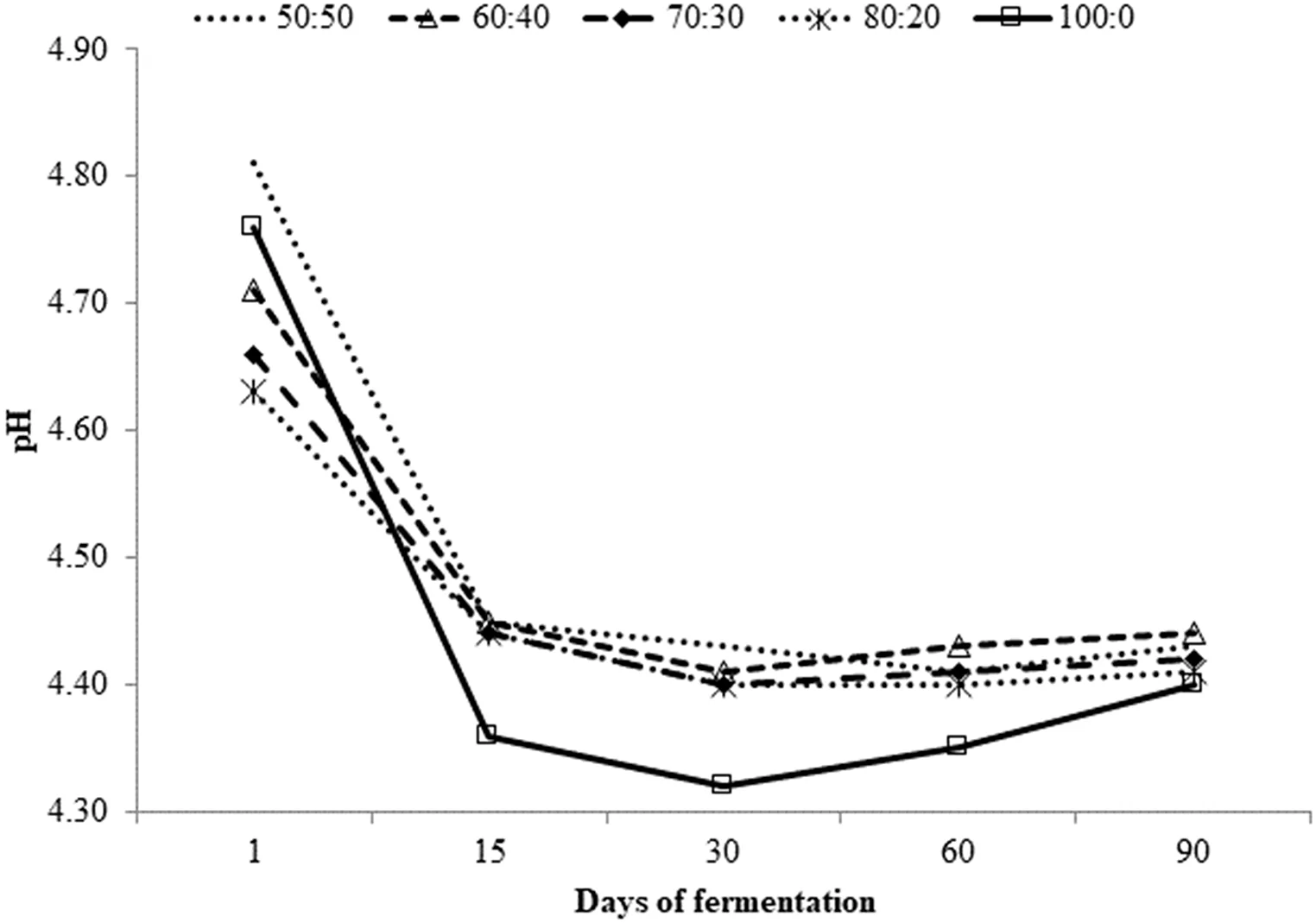
Changes in the pH of total mixed ration silage containing Pornunça (*Manihot* sp.) as a fiber source in different fermentation periods. y (50:50) = [4.7489 – (0.0141x + 0.0001 × ^2^)]; *R²* = 0.80; y (60:40) = [4.6642 – (0.0106x + 0.00009 × ^2^)]; *R²* = 0.77; y (70:30) = [4.6234 – (0.0093x + 0.00008 × ^2^)]; *R²* = 0.80; y (80:20) = [4.6006 – (0.0084x + 0.00007 × ^2^)]; *R*² = 0.83; y (100:0 ) = [4.6899 – [0.0160x + 0.0001 × ^2^)]; *R²* = 0.75

It was found effect of interaction between F: C ratio and the fermentation period on pH (*P* < 0.01; Table 3). For all silages, the highest pH values (*P* < 0.01) were observed after one day of fermentation, and the silage 50:50 presented the highest value (4.82), on the other hand the lowest values (*P* < 0.01) were observed in the silages 70:30 (4.66) and 80:20 (4.63). After 15, 30 and 60 days of fermentation, the silage 100:0 had the lowest (*P* < 0.01) pH values (4.36, 4.32 and 4.35, respectively). At 90 days of fermentation the pH of silage 100:0 was lower (*P* < 0.01) than that of silages 50:50 and 60:40. The pH was affected in a quadratic way by the fermentation period (*P* < 0.001; Table 2; Fig. 1). The equations revealed minimum values of 4.25 (71st day), 4.35 (60th day), 4.35 (58th day); 4.35 (60th day) and 4.05 (80th day) for the silages 50:50, 60:40, 70:30, 80:20 and 100:0, respectively.

**Table 2:** Fermentative profile of total mixed ration silage containing Pornunça (*Manihot* sp.) as a fiber source in different fermentation periods

Day (D)	Forage-to-concentrate ratio (DM basis)							SEM	*P*-value		
	50:50		60:40	70:30	80:20		100:0		F: C	D	F: C×D
	pH										
1	4.82 Aa	4.72 Ac		4.66 Ad	4.63 Ad		4.76 Ab				
15	4.45 Ba	4.45 Ba		4.44 Ba	4.44 Ba		4.36 BCb				
30	4.43 Ba	4.41 Bab		4.40 Cab	4.40 Cb		4.32 Dc	0.01	< 0.0001	< 0.0001	< 0.0001
60	4.41 Bab	4.44 Ba		4.41 BCab	4.40 Cb		4.35 CDc				
90	4.43 Ba	4.44 Ba		4.42 BCab	4.41 BCb		4.40 Bb				
	NH_3_-N (g/kg total nitrogen)										
Mean	40.5b	40.9b		48.2b		47.2b	57.8a	0.23	< 0.0001	0.17	0.14

DM: dry matter

F: C: forage-to-concentrate ratio

NH_3_-N: ammonia nitrogen

SEM: standard error of the mean

Means followed by different uppercase letters in the column and lowercase in the rows are statistically different according to Tukey’s test *P* < 0.05

There was no effect of interaction between F: C ratio and fermentation period on NH_3_-N concentration (*P* = 0.14; Table 3). The concentration of NH_3_-N was affected by the treatments (*P* < 0.01), as the silage 100:0 showed the highest value (57.8 g/kg of total N), with no difference among the others. The fermentation period did not influence the concentration of NH_3_-N (*P* = 0.17).

### Fermentation characteristics, losses, and DM recovery

The pH values were higher (*P* < 0.01) in silages 50:50 and 60:40 when compared to the silage 100:0 which did not differ from the silages 70:30 and 80:20 (Table 4). For NH_3_-N concentrations, there were no significant differences (*P* > 0.05) among silages, showing an average of 45.7 g/kg of TN (Table 3).

**Table 3:** Fermentative characteristics, losses, dry matter recovery and values of organic acids of total mixed ration silage containing Pornunça (*Manihot* sp.) as a fiber source

Item	Forage-to-concentrate ratio (DM basis)					SEM	*P*-value
	50:50	60:40	70:30	80:20	100:0		
pH	4.43a	4.44a	4.42ab	4.41b	4.40b	0.008	0.001
NH_3_-N (g/kg total nitrogen)	49.2	49.5	49.4	30.9	49.5	0.68	0.25
Lactic acid (g/kg DM)	16.8a	17.2a	14.6b	14.1b	13.6b	0.03	< 0.0001
Propionic acid (g/kg DM)	2.26a	2.35a	1.77b	1.88b	0.80c	0.01	< 0.0001
Butyric acid (g/kg DM)	2.01ab	1.89ab	2.07a	2.20a	1.77b	0.01	0.02
Acetic acid (g/kg DM)	4.68a	4.68a	4.32ab	3.95b	2.62c	0.02	< 0.0001
BC (e.mg NaOH)	46.4b	57.7a	63.3a	59.1a	56.0a	2.00	0.0005
Gas losses (g/kg DM)	76.8e	105c	133a	83.3d	114b	0.04	< 0.0001
Effluent losses (kg/t of silage)	5.95c	10.0b	10.5b	23.0a	25.0a	1.64	< 0.0001
DM recovery (g/kg DM)	916a	884c	858e	905b	873d	0.27	< 0.0001

DM: dry matter; NH_3_-N: ammonia nitrogen; BC = buffer capacity; SEM = standard error of the mean

Means followed by different letters on the lines are statistically different according to Tukey’s test *P* < 0.05

The lactic and propionic acids contents were higher (*P* < 0.01) in silages 50:50 and 60:40, with lower values for lactic acid in the silages 70:30, 80:20 and 100:0, while for propionic acid the lowest concentration (0.80 g/kg DM) was observed in the silage 100:0 (Table 3). For butyric acid contents there was a difference (*P* = 0.02) only between silage 100:0 compared to silages 70:30 and 80:20, which presented the highest concentrations (Table 4). For acetic acid, the highest concentrations were obtained in the silages 50:50 and 60:40, and the lowest concentration (2.62 g/kg DM) in the silage consisting exclusively of Pornunça (100:0).

There was a difference in BC (*P* < 0.01) only in the silage 50:50, which showed the lowest value (46.4 e.mg NaOH/100 g DM) when compared to the other silages (Table 3). Regarding fermentative losses, silage 70:30 showed the highest (*P* < 0.01) GL (133 g/kg DM), followed by silages 100:0 (114 g/kg DM), 60:40 (105 g/kg DM), 80:20 (83.3 g/kg DM) and 50:50 (76.8 g/kg DM). As for effluent losses, silages 80:20 (23.0 kg/ton of silage) and 100:0 (25.0 kg/ton of silage), which did not differ (*P* > 0.05) among themselves, presented the highest losses (*P* < 0.01), followed by silages 60:40 (10.0 kg/ton of silage) and 70:30 (10.5 kg/ton of silage) that showed similar losses. On the other hand, 50:50 silage showed the lowest effluent losses (5.95 kg/ton of silage). The DMR showed an inverse behavior to the observed behavior for GL, being the greatest DMR (*P* < 0.01) was found in silage 50:50 (916 g/kg), while the lowest was found in silage 70:30 (858 g/kg; Table 3).

### Microbial population

**Fig. 2:**
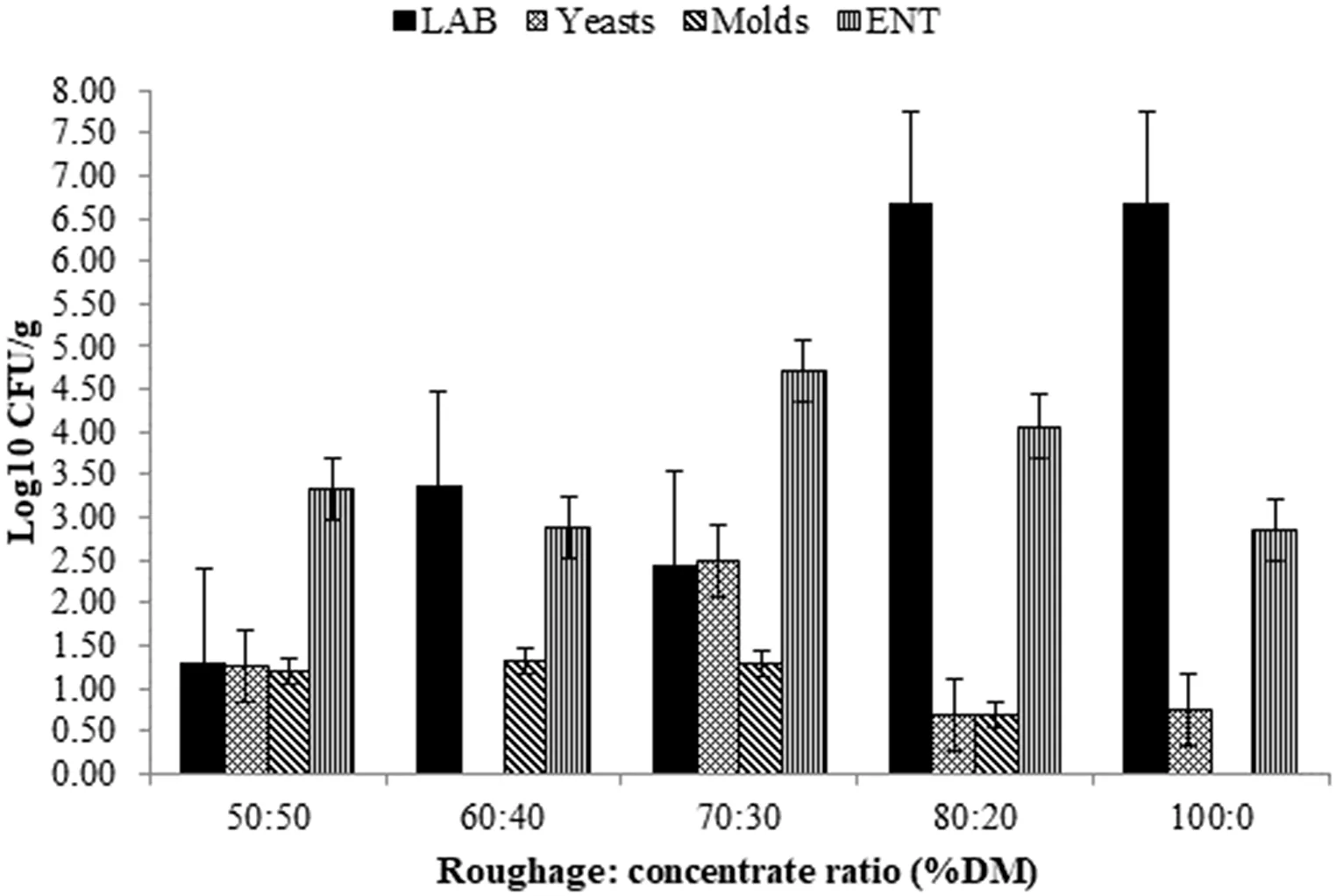
Population of microorganisms of total mixed ration silage containing Pornunça (*Manihot* sp.) as a fiber source (CFU: colony-forming units; DM: dry matter; LAB: lactic acid bacteria; ENT: enterobacteria). (Error bars denote standard errors)

After 90 days of fermentation, LAB were detected in all silages, showing higher counts in the silages 80:20 and 100:0 (Fig. 1). The second most abundant class of microorganisms was the ENT, and the silages 70:30 and 80:20 which presented the highest counts. No molds were found in the silage 100:0, just as yeasts were not found in silage 60:40 (Fig. 2).

### Aerobic stability

**Fig. 3:**
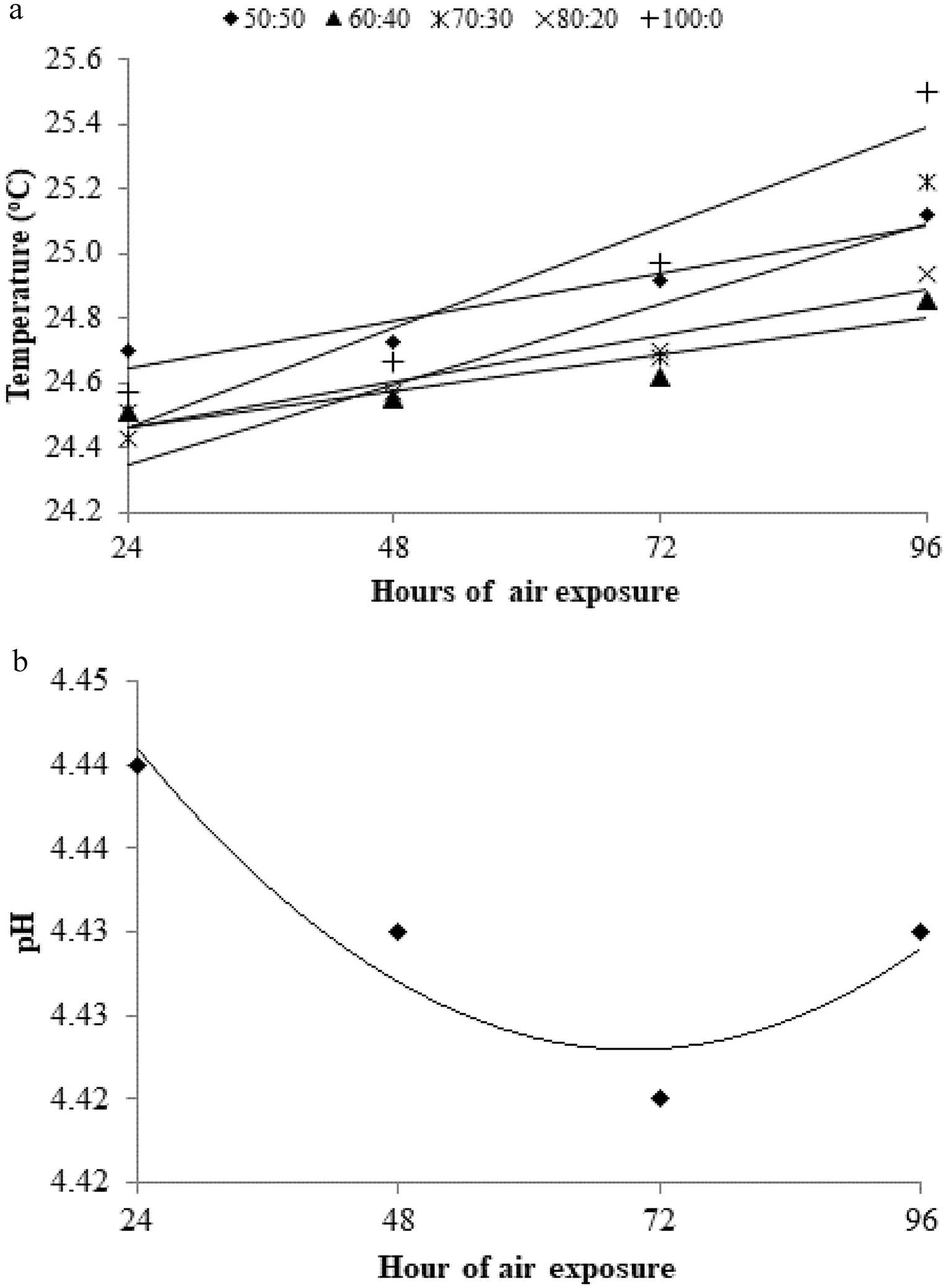
Effect of air exposure time on the temperature and pH of total mixed ration silage containing Pornunça (*Manihot* sp.) as a fiber source. **a**: [50:50 y = 24.50 + 0.006x; *R²* = 0.93; 60:40 y = 24.35 + 0.005x; *R²* = 0.85; 70:30 y = 24.10 + 0.01x; *R²* = 0.86; 80:20 y = 24.32 + 0.006x; *R²* = 0.92; 100:0 y = 24.15 + 0.01x; *R²* = 0.92]. **b**: y = 4.46–0.001x + 0.000008 × ^2^; *R*^*2*^ = 0.90

There was an effect of interaction between F: C ratio and the period of exposure to air on the temperature of the different silages (*P* < 0.01; Table 4). Until 48 h of exposure to air, the silages showed no difference in temperature (*P* > 0.05), but after 72 h, the silage 100:0 showed higher (*P* < 0.01) temperature (24.9 °C) when compared to the silage 60:40 (24.6 °C). In general, the temperature of the silages increased with increasing exposure time to air, especially after 72 h of exposure (*P* < 0.01; Fig. 3a). The silage 100:0 showed the highest temperature (25.5 °C) at 96 h of aerobiosis, and the silage 60:40 was the most stable (24.8 °C).

**Table 4:** Changes in the temperature and pH of total mixed ration silage containing Pornunça (*Manihot* sp.) as a fiber source after 96 h of exposure to air

Hour (H)	Forage-to-concentrate ratio (DM basis)					SEM	*P*-value		
	50:50	60:40	70:30	80:20	100:0		F: C	H	F: C × H
	Temperature (^o^C)								
24	24.71 Ba	24.50 Aa	24.43 Ba	24.58 Ba	24.67 BCa				
48	24.70 Ba	24.55 Aa	24.56 Ba	24.51 Ba	24.57 Ca	0.03	< 0.0001	< 0.0001	< 0.0001
72	24.92 ABab	24.62 Ab	24.68 Bab	24.70 ABab	24.98 Ba				
96	25.12 Abc	24.86 Ac	25.22 Ab	24.94 Abc	25.50 Aa				
	pH								
Mean	4.45a	4.43c	4.43c	4.44b	4.37d	0.002	< 0.0001	< 0.0001	0.60

DM: dry matter; F:C: forage-to-concentrate ratio; SEM: standard error of the mean

Means followed by different uppercase letters in the column and lowercase in the rows are statistically different according to Tukey’s test *P* < 0.05

There was no effect of interaction between F: C ratio and air exposure period on pH value (*P* = 0.60; Table 4). The pH was affected by the F: C ratio (*P* < 0.01), and the silage 50:50 showed highest value (4.45), while the silage 100:0 showed lowest value (4.37). The pH was affected in a quadratic manner with the period of exposure to air, so that the lowest value (4.43) was observed at 63 h of aerobiosis. Given the similarity between the equations, it was decided to present a general equation for all treatments (Fig. 3b).

**Fig. 4:**
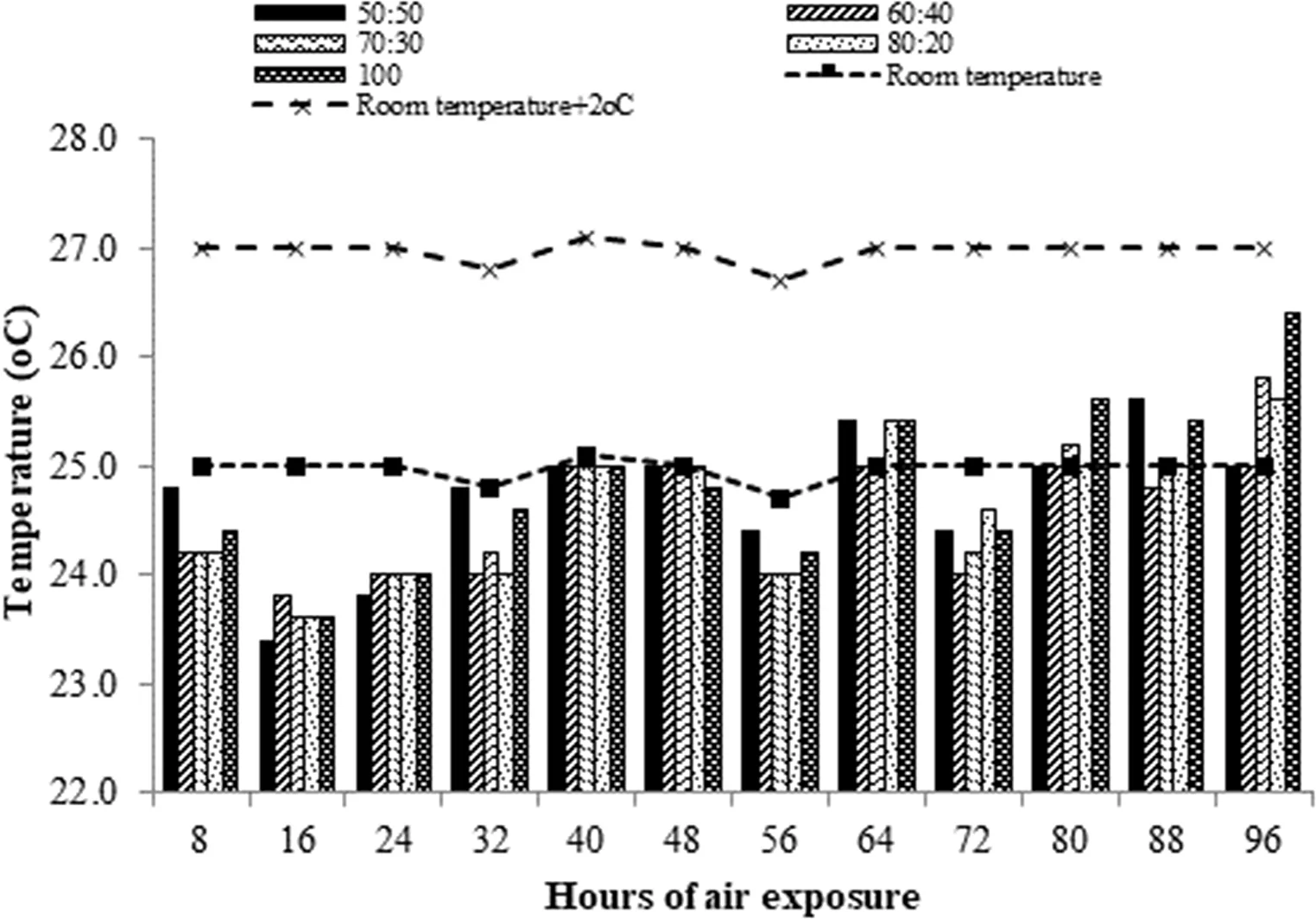
Aerobic stability in total mixed ration silage containing Pornunça (*Manihot* sp.) as a fiber source after 96 h of air exposure

Silages with different F: C ratios did not develop aerobic stability break up to 96 h of air exposure (Fig. 4). The silages 80:20, 50:50, and 100:0 reach temperatures above ambient temperature after 64 h of air exposure, but stability breakdown was not achieved. The silage that had the highest temperature at the end of 96 h was 100:0 (Fig. 4).

### Chemical composition

The DM decreased as the level of Pornunça in the silage increased, with values ranging from 352 to 217 g/kg for silages 50:50 and 100:0, respectively (Table 5). Silages 100:0 and 80:20 showed the highest MM concentrations (*P* < 0.01), on the other hand silages 50:50 and 60:40 showed the lowest concentrations (*P* < 0.01; Table 5).

**Table 5:** Chemical composition of total mixed ration silage containing Pornunça (*Manihot* sp.) as a fiber source

Item (g/kg DM)	Forage-to-concentrate ratio (DM basis)						SEM		*P*-value
	50:50	60:40	70:30	80:20	100:0				
Dry matter (g/kg as fed)	352a	306b	271c	262c	217d	0.45		< 0.0001	
Ash	32.1c	35.2c	61.2b	68.4ab	76.2a	0.23		< 0.0001	
Crude protein	183b	195ab	197ab	204a	185b	0.34		< 0.0001	
NDIP (g/kg crude protein)^a^	195e	251d	296c	328b	377a	0.78		< 0.0001	
ADIP (g/kg crude protein)^b^	57.5d	74.0 cd	88.7bc	108ab	132a	0.70		< 0.0001	
Ether extract	35.9a	36.0a	38.9a	35.9a	22.3b	0.20		< 0.0001	
Total carbohydrates	749a	733ab	703 cd	691d	716bc	0.53		< 0.0001	
Neutral detergent fiber^c^	272e	298d	365c	380b	483a	0.75		< 0.0001	
Acid detergent fiber	188e	210d	265c	288b	375a	0.50		< 0.0001	
Non-fiber carbohydrates	477a	436b	338c	311c	232d	0.84		< 0.0001	
Cellulose	145c	159c	205b	221b	290a	0.51		< 0.0001	
Hemicellulose	83.5b	88.3b	100ab	92.0ab	108a	0.48		0.01	
Acid detergent lignin	43.0c	50.7c	59.8b	67.6b	84.8a	0.19		< 0.0001	
Total digestible nutrients^d^	742a	726b	683c	664d	597e	0.38		< 0.0001	

DM: dry matter; SEM = standard error of the mean

^a^ NDIP: neutral detergent insoluble protein

^b^ ADIP: acid detergent insoluble protein

^c^ Neutral detergent fiber using a heat stable amylase without the use of sodium sulfite and not corrected for ash and protein

^d^ Total digestible nutrients (Undersander et al. 1993)

Means followed by different letters on the lines differ to Tukey’s test *p* < 0.05

The silage 80:20 had greater CP (*P* < 0.01) than silages 50:50 and 100:0, however, it did not differ from the other silages. The NDIP was higher in silage 100:0 and lower in silage 50:50. For ADIP, the silage 100:0 was higher (*P* < 0.01) to silages 50:50, 60:40, and 70:30. Silage 100:0 had the lowest EE (*P* < 0.01), while the others showed no difference (*P* > 0.05; Table 5).

Silage with only Pornunça (100:0) showed the highest (*P* < 0.01) concentrations of NDF, ADF, cellulose, and ADL, while the 50:50 silage presented lowest (*P* < 0.01) concentrations. Lower concentrations of cellulose, hemicellulose, and ADL were also observed in silage 60:40.

In silage 50:50, it was observed the highest (*P* < 0.01) contents of TC and NFC, while in silages 80:20 and 100:0, they were found to be the lowest (*P* < 0.01), respectively. The TDN content decreased with the increase of Pornunça in the silages (*P* < 0.01), ranging from 742 to 597 g/kg DM, for silages 50:50 and 100:0, respectively (Table 5).

### In situ rumen degradability

As the level of Pornunça in the silages increased, there was a reduction in the “*a*” fraction and in the ED of DM (*P* < 0.01; Table 6). For the “*b*” fraction of the DM, highest value (551 g/kg) was found in the silage 70:30 and lowest value (413 g/kg) in the silage 50:50. For the “*c*” fraction of the DM, only the silage 100:0 differed from the others, presenting the highest rate (17.0 g/kg/h). The fraction “*a* + *b*” of DM was higher in silages 60:40 (975 g/kg) and 70:30 (973 g/kg), which also showed greater lag time. The lowest “*a* + *b*” fraction and lag time were found in the silage 100:0.

**Table 6:** Degradation parameters, potential (PD) and effective (ED) degradability of the dry matter (DM) and neutral detergent fiber (NDF) of total mixed ration silage containing Pornunça (*Manihot* sp.) as a fiber source

Item	Forage-to-concentrate ratio (DM basis)					SEM	*P*-value
	50:50	60:40	70:30	80:20	100:0		
	Dry matter						
Fraction *a* (g/kg)	535.8a	488.0b	422.8c	375.7d	292.7e	1.02	< 0.0001
Fraction *b* (g/kg)	413.1e	486.8c	550.7a	513.2b	466.7d	0.60	< 0.0001
*a* + *b* (g/kg)	948.9b	974.8a	973.4a	888.9c	759.4d	1.01	< 0.0001
Fraction *c* (g/kg/h)	11.8b	9.7b	9.9b	11.8b	17.0a	0.00	< 0.0001
*R* ^*2*^	0.96	0.97	0.95	0.98	0.98	-	-
Lag time (h)	8.16c	8.52ab	8.62a	8.38b	7.95d	0.04	< 0.0001
*ED* (20 g/kg/h)	689.1a	647.4b	605.5c	565.7d	500.5e	0.78	< 0.0001
*ED* (50 g/kg/h)	614.7a	560.8b	514.0c	473.5d	407.5e	0.87	< 0.0001
*ED* (80 g/kg/h)	588.9a	540.8b	483.6c	441.6d	372.1e	0.91	< 0.0001
	Neutral detergente fiber^a^						
Fraction *b* (g/kg)	416.8c	410.9c	530.6b	604.1a	428.6c	0.84	< 0.0001
Fraction *c* (g/kg/h)	46.4a	28.4b	24.0c	15.0d	27.1b	0.00	< 0.0001
*U* (g/kg)	583.2a	589.1a	469.4b	395.9c	571.3a	0.84	< 0.0001
*R* ^*2*^	0.98	0.99	0.98	0.99	0.99	-	-
Lag time (h)	6.80d	7.28c	7.70b	8.30a	7.37c	0.04	< 0.0001
*ED* (20 g/kg/h)	291.1a	239.8c	289.5a	259.5b	246.7c	0.25	< 0.0001
*ED* (50 g/kg/h)	200.5a	147.8c	172.2b	139.8d	150.7c	0.13	< 0.0001
*ED* (80 g/kg/h)	152.9a	106.8c	122.5b	95.7d	108.5c	0.09	< 0.0001

*a*: soluble fraction; *b*: potentially degradable insoluble fraction; *c*: fraction *b* degradation rate; *a* + *b*: degradable fraction; *U*: undegradable fraction; SEM: standard error of the mean

^a^ Neutral detergent fiber using a heat stable amylase without the use of sodium sulfite and not corrected for ash and protein

Means followed by different letters in the rows are statistically different according to Tukey’s test *P* < 0.05

Regarding the DEG of NDF, silage 80:20 showed highest value (604 g/kg) for the fraction “*bs*”, while the silages 50:50, 60:40 and 100:0 had lowest values (Table 6). The fraction “*c*” was lower in the silage 80:20 (15.0 g/kg/h) and higher in the silage 50:50 (46.4 g/kg/h); being also found in the silages 80:20 (8.3 h) and 50:50 (7.28 h) the highest and lowest lag time for NDF, respectively. Higher fractions “*U*” of NDF, were found in silages 50:50, 60:40 and 100:0, while the lowest “*U*” was observed in the silage 80:20. Considering kp of 20 g/kg/h, ED of NDF was higher in silages 50:50 and 70:30, and lower in the silages 40:60 and 100:0. Considering kp of 50 g/kg/h and 80 g/kg/h, ED of NDF was higher in silage 50:50 and lower in silage 80:20.

## Discussion

### Fermentation profile, losses, DMR and microbial population

Based on the silage characteristics, the fermentative process can be considered efficient, as indicated by low pH values, NH₃-N content below 100 g/kg of TN, and the predominance of lactic acid (Bueno et al. 2020). The resistance to further pH reduction may be associated with the high buffering capacity (46.4 to 63.3 mEq/100 g of DM) and the average CP content (160 g/kg) of the silage. Nevertheless, the observed pH range (4.40–4.44) falls within the intermediate range (4.3–4.5) reported for well-preserved silages (McDonald et al. 1991). Possibly, the adequate availability of fermentable carbohydrates favored the activity of LAB, resulting in appropriate final pH values and indicating efficient fermentation and satisfactory silage quality.

It is well known that the microbial population plays an important role in defining the fermentative profile of silage, as the predominance of specific groups of microorganisms determines the rate of pH decline, the type and amount of organic acids produced, and, consequently, the stability and quality of the ensiled material. Silages with a higher abundance of LAB tend to exhibit more efficient fermentation, characterized by greater lactic acid production and a rapid decline in pH, which inhibits the growth of undesirable microorganisms such as enterobacteria, clostridia, yeasts, and filamentous fungi (Muck, 1991; Pahlow, 2003).

Although the differences were minimal, statistical analyses revealed significant differences between silages for pH, indicating that the silages 80:20 and 100:0 had the lowest values. However, these silages presented the lowest concentrations of organic acids, mainly lactic acid. Usually, this behavior is unexpected because lactic acid is the main acid responsible for lowering the pH of silage. Another point that deserves to be highlighted is that the silages 80:20 and 100:0 were those that presented the higher LAB counts (Fig. 2), however, with the lower lactic acid concentrations, which seems paradoxical behavior. These findings, at least for lactic acid concentrations, can be explained as stated by Lindgren et al. (1990), in which some strains of LAB are able to use lactic acid anaerobically when glucose is limiting.

In general, the silages with a higher proportion of concentrate had a higher concentration of organic acids, especially lactic acid and acetic acid. This behavior was expected, because the concentrate is a substrate to be fermented by the silo microbiota. However, the higher concentration of these acids did not reflect in lower pH value. This behavior was unexpected. The higher lactic acid values of silages with higher amounts of concentrate (50:50 and 60:40) may reflect a possible increase in the amount of soluble carbohydrates (data not analyzed), the main substrate used by LAB for lactic acid production (Zhao et al. 2021). The possible conversion of lactic acid into propionic acid and 1-propanol by the microbiota during ensiling (Kung Jr et al. 2018) may explain the higher concentrations of propionic acid in the silages 50:50 and 60:40. All silages showed propionic (< 5.0 g/kg DM), acetic (< 20.0 g/kg DM) and butyric (< 3.0 g/kg DM) acid values below the maximum recommended limit for obtaining good-quality silage (Kung Jr et al. 2018).

Efficient preservation of TMRs with minimal losses during the fermentation period is important in providing essential nutrients to ruminants (Ning et al. 2017). High moisture content and the development of yeasts and ENT during ensiling are factors that can promote higher losses and, consequently, decreased silage DM recovery (Gusmão et al. 2018). These factors possibly influenced the losses found in this study, as the highest EL were observed in silages with higher moisture contents (80:20 and 100:0) and higher GL in the silage 70:30, which numerically presented higher counting of yeasts and ENT (Fig. 2).

In TMRs it is common to find DMR higher than 950 g/kg (Hu et al. 2015; Miyaji et al. 2017; Restelatto et al. 2019) associated to high DM content (400 to 500 g/kg) and high density. In this study, the highest DMR which was found in silage 50:50 (916 g/kg), may be associated to the higher DM content (352 g/kg; Table 2) of this silage, however, this value is still below what is considered optimal (> 950 g/kg), for TMR silages (Restelatto et al. 2019).

Silage losses always occur even in a well-managed conservation system used for silage production. However, one of the main objectives of the producers is to reduce losses in the whole ensiling process. The level of losses and the quality of the silage process can be affected by many factors (Borreani et al. 2018). Gas and effluent losses are directly influenced by forage moisture content (Sobral et al. 2024). Thus, exclusively ensiling *Manihot* sp negatively affects fermentation characteristics, increasing GL and EL, and reducing DMR. The reduction of these losses is mainly associated with the increase in DM content as can be observed in silage with a higher proportion of concentrate (50:50) in which it presented the highest DMR. From a practical point of view, this has important implications since DMR refers to the amount of DM that is preserved and available for feeding after the silage process, compared to the initial DM of the material. The association of ingredients used in the preparation of TMR was beneficial since it contributes to reducing nutrient losses through leaching, improving the nutritional value of TMR.

The greater development of LAB in silages 80:20 and 100:0 may reflect the optimal environment for growth of these microorganisms, which was expected in silages with higher amount of concentrate (50:50 and 60:40), considering a possible increase in the amount of substrate for the development of the microbiota (Gusmão et al. 2018). But other factors such as temperature and moisture are also important in the development of microorganisms (Kondo et al. 2015). High DM content during ensiling can slow microbial multiplication (Mcdonald et al. 1991; Qiu et al. 2014; Yin et al. 2017). This may have contributed to the lower development of LAB in silages with higher amounts of concentrate (50:50, 60:40, and 70:30) but showed no effect regarding to ENT. The pH of the silages close to 4.5 may have contributed to the development of ENT (Wang et al. 2016).

### Aerobic stability

The evaluation of silage aerobic stability provides a basis for determining the silage quality, regarding the preservation of nutrients and the counting of fungal spores and toxins during a given period of exposure to air (Yuan et al. 2015). Aerobic spoilage can be seen by temperature increase, pH changes, and increase in yeast count, with temperature increase by 2 °C above room temperature being the most adopted to assess spoilage (Wang et al. 2016). In the present study, none of the silages presented a breakdown of aerobic stability, i.e., the temperature did not exceed the limit of 27 °C. This result is important because silage is inevitably exposed to air and susceptible to aerobic deterioration when fed to animals or when subjected to long-distance transport.

Silages with high moisture tend to have lower stability when exposed to oxygen (Hao et al. 2015), which may have favored the silage 100:0 to have the highest temperature at 96 h of aerobiosis. In addition, aerobic deterioration is favored by high LAB and yeast populations associated with high amount of substrates (Hao et al. 2015; Yuan et al. 2015; Wang et al. 2016). The significant increase in silage temperature with of silage 70:30 at 96 h of aerobic exposure, may have been due to the development of yeasts during ensiling, as well as the reverse may explain the lower temperature in the silage 60:40 (Fig. 2).

In the present study, the pH of the silage decreased after air exposure, reaching the lowest value around 60 h of aerobic exposure, and after this period, pH increased in all silages. After exposure to air, silage deterioration can be carried out by aerobic bacteria, yeasts, and molds (Yuan et al. 2015). With the activity of these microorganisms soon after air exposure, there is a reduction in lactic acid (substrate), and consequently an increase in pH; however, if the decrease in lactic acid is slow, relative to the production of acids by aerobic microorganisms, a decrease in pH may occur at the beginning of air exposure (Wang et al. 2016).

### Chemical composition

The chemical composition of TMR can be influenced by several factors, among the main ones are the balancing of the diet, the quality of the mixture and the development of the fermentative process (Bueno et al. 2020; Li et al. 2021). In this study, the different F: C ratios of the silages promoted variations in the concentrations of some nutrients. The reduction of DM in the silages with increasing proportion of Pornunça, was expected because the concentration of DM in that forage is lower than in the other ingredients. This result was observed for the concentrations of other nutrients such as EE and TC (Tables 1 and 2).

The rations were formulated to be iso-protein with an average of 168 g CP/kg DM (Table 1) and, after 90 days of fermentation, they presented CP above 180 g/kg DM (Table 2). This fact, associated with the superiority of 80:20 silage compared to silages 50:50 and 100:0, can be explained by consumption of soluble carbohydrates by the microbial activities inside the silo, increasing the concentration of more complex nutritional constituents, based on the DM of the silage (Miyaji et al. 2017). Li et al. (2021) observed similar results studying TMR and found increase of CP content after ensiling the rations, and associated this response to the efficient fermentative process, thus conserving the nutritional content of the rations. In turn, Chen et al. (2015) attributed the increase of CP content to a concentration effect with increasing DM and increasing microbial derived CP.

The breakdown of protein and conversion to non-protein nitrogen (NPN) also occurs in TMR silages, however the extent of these processes is limited by the usually high DM concentration of the silages and ingredient sources (Bueno et al. 2020). Hao et al. (2015) evaluated the effects of moisture content (400, 450, and 500 g/kg of moisture) and reported higher moisture content increased the concentrations of NPN, free amino acids (AA), and NH_3_-N during fermentation. This probably explains the fact that silage 80:20 (262 g/kg DM as fed) has the highest concentration of CP.

Research has shown that TMR silage increases the solubility of protein and the proportion of NPN (urea, free amino acids, small peptides, nitrates and nitrites, etc.) (Lazzari et al. 2021; Bueno et al. 2020). It is known that the determination of CP by the Kjeldahl method does not separate the protein-nitrogen from the nonprotein nitrogen compounds. Thus, one of our hypotheses is that the probable increase of NPN in the TMR was counted as protein, because the determination method slightly overestimates the true protein content because the NPN is included in the total nitrogen result.

The increase in fiber-bound protein fractions (NDIP and ADIP) in silages with higher proportion of Pornunça may be due to the higher concentrations of these fractions in the forage (Table 1). Lower NDIP and ADIP fractions are desirable because they characterize the availability of protein to be digested during its passage through the animal’s digestive tract (Carvalho et al. 2021). Thus, silages with a higher proportion of these fractions tend to have lower total protein digestibility (Campos et al. 2017).

The MM, NDF, ADF, hemicellulose, cellulose, and ADL contents in silages with higher level of forage, reflected their composition before ensiling. In the present study, there was a reduction in the fiber fraction for most silages when compared to the feed before ensiling, except for the silage with F: C 100:0 which maintained almost the same value of ADF content (Tables 1 and 2). During the ensiling process, degradation of the NDF of the feeds can occur because of the low content of water-soluble substrates (simple sugar and starch) and the development of undesirable microorganisms (Ning et al. 2017). In studies of Wang et al. (2016) and Ning et al. (2017), reductions in starch and hemicellulose concentrations were found after ensiling, which suggested that these nutrients could serve as substrates for microbiota to produce acids. This fact may explain the response observed in the present research.

The silage 80:20 showed the greatest reduction in TC, when compared to the values before ensiling. This may be associated with the increase in CP and EE contents observed in this silage. The reductions in cellulose and hemicellulose in the silages 50:50 and 60:40 (comparing Tables 1 and 2) explain the largest NFC contents of these silages, when compared with the values before ensiling. The higher concentration of ADF in the silage 100:0 is directly linked to the higher lignin content, as this phenolic compound is one of the main substances that makes up this fibrous fraction, and responsible for reducing feed digestibility (Van Soest et al. 1991). The decrease in NFC of the silages with increasing participation of Pornunça can be explained by the increase in the NDF fraction in these silages.

With the increase of concentrate in the rations, the fractions of lower digestibility (ADF, NDIP) reduced, promoting an increase in TDN concentration, which explains the higher concentration of this fraction found in silage 50:50 (Undersander et al. 1993). Besides, losses through effluents during ensiling may promote decrease in sugars and proteins (Mcdonald et al. 1991), which contributes to lower TDN concentrations.

### In situ rumen degradability

The synchronization in the availability of nutrients to the ruminal microbiota is essential for its multiplication, and thus for rumen degradability (Costa et al. 2020). Concentrates improve the nutritive value of TMR, and with the fermentative process developed properly, this feed can have an increase in solubility, increasing the availability of nutrients for the rumen microbiota (Bueno et al. 2020; Du et al. 2020).

The higher soluble fraction and higher *ED* of DM values of the silage 50:50, are probably associated with the higher soluble fraction of the concentrate ingredients when compared to the Pornunça. In addition, during ensiling, the fermentative process may have favored the increase of this fraction, from the action of microbial enzymes (Miyaji et al. 2017). With the increase of the soluble fraction of DM, a reduction of the “*P”* fraction can be expected, due to part of the total degradable fraction (*a* + *b*) having already been solubilized, which explains the lower *b* fraction of the silage 50:50. The silage 100:0 had a high concentration of ADIP and ADL, which probably contributed to this silage having lower *a* + *b* fraction and, consequently, lower *ED* of DM considering all *k*. This corroborates the reduction of TDN in silages with higher proportion of Pornunça.

Lignin reduces cell wall digestibility by acting as a barrier to the access of cellulolytic enzymes produced by microorganisms, reducing rumen degradation (Cao et al. 2013). This may be considered the main contributing factor that silages with higher amount of Pornunça, showed lower *ED* of NDF. The ensiling process may contribute to the increase of fiber degradability, through the action of enzymes of microorganisms (Bueno et al. 2020), which may have favored the *ED* of NDF of silage 70:30. The nutritive value of the silage 50:50 may have contributed to microbiota development, and consequently fiber degradation, as well as the low lignin content.

Therefore, the hypothesis that incorporating concentrated feedstuffs to Pornunça for silage making as TMR would promote beneficial changes on the fermentative profile of silages, reducing losses and allowing the preservation of the nutritional value of silages was confirmed by the analyzed data.

## Conclusion

Among the TMR silages, the 50:50 forage-to-concentrate ratio provided the best balance of fermentation quality, nutrient preservation, and ruminal degradability, making it the most suitable option for smallholder lamb production in semiarid regions.

## Data Availability

The datasets generated and analyzed during the current study will be provided upon reasonable request from the corresponding author.
